# Observational Study on the Distribution of Cat Blood Groups in a Non-Pedigree Population in Luanda, Angola

**DOI:** 10.3390/vetsci12040357

**Published:** 2025-04-11

**Authors:** Ana C. Silvestre-Ferreira, Hugo Vilhena, Ana C. Oliveira, José R. Mendoza, Maria Garcia Aura, Josep Pastor

**Affiliations:** 1 Department of Veterinary Sciences, University of Trás-Os-Montes and Alto Douro (UTAD), 5000-801 Vila Real, Portugal; 2 Animal and Veterinary Research Centre (CECAV), University of Trás-Os-Montes and Alto Douro (UTAD), 5000-801 Vila Real, Portugal; hcrvilhena@hotmail.com; 3 Associate Laboratory for Animal and Veterinary Science—AL4AnimalS, 1300-477 Lisboa, Portugal; 4 Center for Investigation Vasco da Gama (CIVG), Department of Veterinary Sciences, Escola Universitária Vasco da Gama (EUVG), 3020-210 Coimbra, Portugal; 5 Department of Veterinary Clinics, School of Medicine and Biomedical Sciences, ICBAS-UP, University of Porto, 4050-313 Porto, Portugal; 6 Casa dos Animais Veterinary Clinic, Luanda 298, Angola; olivecris@hotmail.com (A.C.O.); joseriveromendoza@gmail.com (J.R.M.); rudysnel1992@hotmail.com (M.G.A.); 7 Department of Animal Medicine and Surgery, Universitat Autònoma de Barcelona (UAB), Bellaterra, 08193 Barcelona, Spain; josep.pastor@uab.cat

**Keywords:** Angola, blood groups, blood types, cat, feline, neonatal isoerythrolysis, transfusion reactions

## Abstract

Post-transfusion reactions can occur in animals because they present alloantibodies that do not require prior sensitization. Therefore, blood typing is necessary to reduce the risk of hemolytic reactions, but so far, no studies have been carried out to establish the incidence of common blood types in cats from Angola. Therefore, the objective of this observational study was to assess the occurrence of cat blood types in a non-pedigree feline population of Luanda province in Angola, a country in Sub-Saharan Africa, and to determine the risk of a mismatched transfusion and of neonatal isoerythrolysis (NI). Of the 127 cats tested (71 males and 56 females), including diseased and healthy cats with ages ranging from 8 months to 17 years, 94.9% were type A and 5.1% were type B. No type C cats were found. The calculated risk of a mismatched transfusion was 9.64%, and the proportion of mating risk for NI was 4.82%. Our results, although similar to those reported globally, differ from data described in other studies on the African continent. According to the results, blood typing is recommended prior to transfusion or matting.

## Simple Summary

The blood group ABC is the most clinically important in cats because of the existence of naturally occurring alloantibodies responsible for post-transfusion hemolytic reactions and neonatal isoerythrolysis. Cats express antibodies against the blood type they lack. The occurrence of cat blood types varies geographically and between breeds. The aim of this study was to determine the occurrence of cat blood types in a non-pedigree cat population of the Luanda province in Angola (Sub-Saharan Africa) and to assess the risk of a mismatched transfusion and neonatal isoerythrolysis. Of the 127 cats tested (71 males and 56 females), 94.9% were type A, and 5.1% were type B. No type C cats were found. The risk of a mismatched transfusion was 9.64%, and the proportion of mating risk for neonatal isoerythrolysis was 4.82%. According to the results, blood typing is recommended prior to transfusion or matting.

## 1. Introduction

The most important blood group system in the cat is the ABC (former AB), in which cats are classified as type A, B, or C [1,2]. Although cat blood types are known to vary geographically and within breed [3], type A is the most prevalent worldwide, especially among the non-pedigree cat population [4], and types B and C are reported as rare [5,6,7,8]. Variations in blood type distribution can also be found within countries [5,9,10,11].

Cats express alloantibodies (hemagglutinins and hemolysins) against the blood type they lack, as they do not require prior sensitization by transfusion, pregnancy, or other blood products [12,13]. Naturally occurring alloantibodies are responsible for post-transfusion hemolytic reactions that can lead to animal death if a type B cat receives type A or C blood [14,15] and also for the occurrence of neonatal isoerythrolysis (NI) in type A or C kittens when they are born to a type B queen and absorb mother’s colostrum within the first 24 h after birth [16,17,18].

As previously reported, all type B cats over three months of age possess high titers of naturally occurring alloantibodies (IgM) against blood type A, but only one-third of type A cats have measurable titers of naturally occurring anti-B alloantibodies (IgG); however, no alloantibodies have been found in blood type C cats [12,19,20]. Blood-type antigens are related to the presence of the gangliosides, N-acetylneuraminic acid (NeuAc) and N-glycolylneuraminic acid (NeuGc), on the surface of erythrocytes, which are expressed differently in different individuals [21,22,23], and are coded by Cytidine monophospho-N-acetylneuraminic acid hydroxylase (CMAH) mutations [24,25,26].

Several reports on crossmatch incompatibilities and post-transfusion reactions in well blood-typed transfusions lead to the suspicion of new blood types [27,28], such as Mik [29], and studies with crossmatch detected five new feline erythrocyte antigens outside the ABC system [30], but with clinical relevance yet to be clarified [30,31]. Despite these, the ABC is still the most clinically relevant blood group system in cats, and post-transfusion reactions, or NI, occur related to blood type A and B frequency, with the severity of the reactions depending on the antibody titers [20]. The risk of a life-threatening post-transfusion reaction (major transfusion reaction—MTR) is defined as the risk of a mismatched transfusion (MT) between a type A or C donor and a type B recipient. A minor transfusion reaction (mTR) can reduce the erythrocytes’ life span and results from type B donors and type A recipients [32]. The safety and efficacy of blood transfusion are then ensured by typing the blood of donor and recipient animals within the ABC blood group system. To prevent post-transfusion reactions not related to this blood group system, a crossmatch test should be performed [9,13,33].

To the best of our knowledge, there are no previous studies on the occurrence of the ABC blood group system in the cat population in Luanda, Angola, and the risks of a mismatched transfusion or mating are unknown. Considering the previous research on feline blood groups in other countries and continents, a higher prevalence of type A and a low prevalence of type B and C is expected; however, due to the scarcity of information regarding feline blood groups in the African continent, the results could be different than those previously reported. For that reason, the aim of this work was to determine the occurrence of blood types A, B, and C in a privately owned non-pedigree cat population from Luanda, a province of Angola, a country in Southern Africa, and to assess the risk of a mismatched transfusion and NI in this feline population.

## 2. Materials and Methods

Whole blood samples were collected into EDTA tubes from non-pedigree cats that attended the veterinary medical care center Clínica Veterinária Casa dos Animais, Luanda, between 21 June and 24 November. Cats that needed blood typing for blood transfusion were included in the study. Diseased cats that needed blood work as part of their diagnostic plan and healthy cats from which blood was collected for health check-ups, determination of FIV and FeLV infection status, or as part of the pre-anesthetic evaluation for elective surgeries were also enrolled in the study. The animals were selected randomly. No samples were collected on purpose for the study, and blood typing results were provided to all cat owners as part of their pet clinical information. No cat had a history of previous blood transfusion. All owners gave informed written consent for the use of surplus blood samples for blood typing (10 μL). From each animal, data on sex and age were collected. Samples were kept refrigerated at 4 °C until blood typing was performed on the same day of blood collection. Blood typing was performed with the immunochromatographic strip technique (QuickTest BT A + B, Alvedia, Limonest, France) based on the use of monoclonal antibodies against A/B antigens in cats, following the manufacturer’s instructions.

### Statistical Analysis

SPSS Statistics software version 22 was used for statistical analysis. Blood type frequencies were calculated as percentages. Fisher’s exact test was used to compare statistically significant differences between genders. Statistically significant differences were set at *p* value < 0.05.

The NI risk was estimated according to the Hardy–Weinberg equilibrium and to the equation (*p*2)(q2) + 2*p*q(q2). In this equation, q = b allele frequency; *p* = 1 − q. The MT risk was estimated by adding the MTR risk to the mTR risk. Type AB cats were not identified in this study; the risks of the major and minor transfusion reactions and NI were, therefore, calculated by the type-B frequency multiplied by the type-A frequency [32].

## 3. Results

Of the 127 blood samples collected, 71 were from males and 56 from female cats aged between 8 months and 17 years. Overall, the prevalence of blood types A and B was 94.9% (n = 123) and 5.1% (n = 4), respectively. No type C cats were found (Table 1). There was no statistical association between the frequency of blood type and sex (*p* = 0.319). Based on the blood type frequencies, the risk of an MT was 9.64%, and the proportion of mating risk for NI was 4.82%.

## 4. Discussion

Blood typing of the donor and of the recipient is of utmost importance before blood transfusion in cats to reduce the risk of hemolytic reactions. Geographic and breed variations in the prevalence of feline blood types are well documented in the veterinary literature [3,4,5,6,7,8,9,10,11]; however, to the authors’ best knowledge, no information is available regarding the prevalence of common blood types in cats from Angola. For this reason, this investigation aimed to determine the prevalence of blood types in a non-pedigree feline population of Luanda, Angola (Sub-Saharan Africa), and to assess the risk of a mismatched transfusion and NI. In this study, most cats tested were type A (94.9%), and 5.1% were type B (no cat tested type C), and the risk of a mismatched transfusion was estimated to be 9.64%, and the proportion of mating risk for NI was estimated to be 4.82%.

To our knowledge, only one study assessed cat blood types in the African continent. In Maiduguri (Nigeria), blood type A was found to be the most common with 88% of the tested animals, 12% were of type B, and no type C cats were detected [34]. In our study, held in Luanda (Angola), blood type A frequency was higher than that of Nigeria, but overall, our results are similar to those previously described worldwide, with blood type A being the most frequent [4]. The occurrence of blood type B varies mostly with the animal’s breed, but geographical variation within non-pedigree cats is well documented between countries, ranging from 36% and 25% in Australia [32] and Turkey [7], respectively to 1% or 0% in the USA [10] and Hungary [35]. Variation is also observed within countries. In the United Kingdom, type B occurrence may vary from 30.5% in the southern to 7.9% in the northern country [36,37], and in Portugal, a frequency of 4.4% was observed in the northern country [5] and a frequency of 2.1% in Lisbon region [11], highlighting the significance of understanding feline blood groups. Our results regarding blood type B are significantly different from those from Nigeria (12%) [34] but are comparable to the majority of studies and locations.

In most geographical locations, type C blood in non-pedigree cats is rare, typically ranging from 0% to 1% [8,9,10,11,38,39,40]; however, in Northern Portugal, a frequency of 6.3% of type C cats was found [5]. Our findings align with previous global studies and those reported in Nigeria (0%) [34]. Also, similarly to the majority of studies regarding sex, we did not find a statistically significant association with blood type occurrence; however, we have only four animals with type B blood (3 females and 1 male) [33,41,42]. Nonetheless, Maryam et al. (2014), in Nigeria, found a significant difference in the frequency of type B blood between the females and the males [34].

Blood typing and knowledge of feline blood type occurrence are of utmost importance in preventing post-transfusion reactions in mismatched blood transfusions or even NI. In our study, based on the blood type frequencies, the risk of an MT was 9.64%. This is mainly because of the high titers of naturally occurring anti-A alloantibodies with strong hemolyzing and agglutinating activity present in type B cats. Naturally occurring alloantibodies can lead to fatal transfusion reactions, premature destruction of transfused red blood cells [15,43], and NI in type A or C kittens born to type B queens due to the presence of anti-A antibodies in the colostrum [16,17]. Naturally occurring alloantibodies are directed against the glycolipid antigenic determinants of the erythrocyte membrane. Blood types A and B are inherited as simple autosomal Mendelian traits, with A being dominant over B. Type C (former AB) is allelic to A and B. The glycolipid antigenic determinants on the erythrocyte membrane for the A, B, or C phenotype are NeuAc and NeuGc. Blood types are determined by mutations in CMAH, the enzyme responsible for converting NeuAc to NeuGc [24,25,26]. These are expressed in various combinations among different individuals. Type B cats express only NeuAc, type A cats express both NeuGc and NeuAc-NeuGc disialogangliosides, while type C cats express equal amounts of disialogangliosides containing NeuAc or NeuGc [22,23]. A newly identified alloantibody named Mik was recently discovered against a common red blood cell antigen. The clinical significance of anti-Mik alloantibodies was demonstrated through an acute hemolytic transfusion reaction that occurred after a mismatched transfusion of a Mik-positive cat to a Mik-negative recipient [29]. A 15% prevalence of non-ABC blood type incompatibilities in first transfused cats, likely associated with naturally occurring Mik-alloantibodies, was found [27]. More recently, a study including 1228 crossmatches with blood from 258 type A cats identified seven new naturally occurring alloantibodies outside the ABC blood group system [30]. Currently, there are no commercial methods available for blood typing outside the ABC blood group. The recognition of a significant prevalence of naturally occurring non-ABC incompatibilities justifies the need for a crossmatch before every red blood cell transfusion in cats.

Kittens with blood group A, born to a queen with blood group B, will receive anti-A alloantibodies through the colostrum and may develop NI. This can lead to fatal intravascular hemolysis within the first few days of life. Surviving kittens may exhibit necrosis of the tail tip [16,43]. Conversely, kittens with blood group B, born to a queen with blood group B, will remain unaffected [43]. Although there are several reports [16,17,18,44], the overall incidence of NI in feline populations is unknown. However, it should be lower in non-purebred cats than in breeds with a high prevalence of type B blood [44]. Knowing the prevalence of blood type B and using the Hardy–Weinberg equilibrium, it is possible to estimate the risk of NI in a feline population when excluding blood type C [32,44]. In this study, no blood type C cats were found, and the proportion of mating risk for NI was 4.82%. Although not too high this result reinforces the need for premating compatibility testing by blood typing and/or crossmatch [45].

The risk of MT and NI are dependent on the frequency of type B blood and are relatively low when compared to other geographical locations such as Sydney (Australia) (45.3% and 23% respectively) [32] but comparable to Barcelona (Spain) or Montreal (Canada) where a percentage of 9.5% for MT and 4.8 for NI were found [41].

In this study, the calculated risk of MT only considers the ABC blood group system and does not take the Mik phenotype or others into account. Whether or not the risk reported here corresponds to the clinical practice reality is not possible to assess since not all post-transfusion reactions are reported and can be prevented by blood typing or crossmatch. Regarding limitations, this is a local study conducted on a relatively small number of only non-pedigree cats. Therefore, caution is recommended when interpreting the results and when these findings are extrapolated to other geographic areas. The immunochromatographic test used has shown high sensitivity and specificity in identifying feline blood types A, B, and C [46,47]. However, the absence of back-typing for B and C samples might also represent a limitation of this study.

## 5. Conclusions

To the best of our knowledge, this is the first study on the ABC blood group system in cats carried out in Angola. The results underscore the value and importance of regional studies in identifying the varying prevalences of cat blood types. Blood typing should be regarded as an essential test for cats of all origins to ensure safe and effective blood transfusions and to prevent neonatal isoerythrolysis. In Luanda, the prevalence of type B cats, although relatively low, is associated with non-negligible risks of MT and NI, confirming, in the studied area, the importance of blood typing prior to any blood transfusion or even mating.

## Figures and Tables

**Table 1:** Demographic characteristics and blood typing results of the 127 cats tested, and risks for major transfusion reaction (MTR), minor transfusion reaction (mTR), mismatched transfusion (MT), and neonatal isoerythrolysis (NI).

	Nº of Cats	A N (%)	B N (%)	C N (%)	MTR (%)	mTR (%)	MT (%)	NI Mating Risk (%)
Male	71	70 (98.6)	1 (1.4)	0	-	-	-	-
Female	56	53 (94.6)	3 (5.4)	0	-	-	-	-
Total	127	123 (94.9)	4 (5.1)	0	4.82	4.82	9.64	4.82

## Data Availability

Data will be made available upon reasonable request to the corresponding author.

## Abbreviations

The following abbreviations are used in this manuscript:

CMAH	cytidine monophospho-N-acetylneuraminic acid hydroxylase
MT	mismatched transfusion
MTR	major transfusion reaction
mTR	minor transfusion reaction
NeuAc	N-acetylneuraminic acid
Neu-Gc	N-glycolylneuraminic acid
NI	neonatal isoerythrolysis
